# The phenotypic spectrum of dihydrolipoamide dehydrogenase deficiency in Saudi Arabia

**DOI:** 10.1016/j.ymgmr.2021.100817

**Published:** 2021-10-23

**Authors:** Anar Alfarsi, Majid Alfadhel, Seham Alameer, Amal Alhashem, Brahim Tabarki, Faroug Ababneh, Ahmed Al Fares, Fuad Al Mutairi

**Affiliations:** a Genetics & Precision Medicine Department, King Abdulaziz Medical City, Ministry of National Guard-Health Affairs (NGHA), Riyadh, Saudi Arabia; b King Saud bin Abdulaziz University for Health Sciences, Ministry of National Guard-Health Affairs (NGHA), Riyadh, Saudi Arabia; c King Abdullah International Medical Research Centre, Ministry of National Guard-Health Affairs (NGHA), Riyadh, Saudi Arabia; d Department of Pediatrics, King Abdulaziz Medical City, Ministry of National Guard-Health Affairs (NGHA), Jeddah, Saudi Arabia; e Division of Genetics, Department of Pediatrics, Prince Sultan Military Medical City, Riyadh, Saudi Arabia; f Department of Anatomy and Cell biology, college of Medicine, Alfaisal University, Riyadh, Saudi Arabia; g Division of Neurology, Department of Pediatrics, Prince Sultan Military Medical City, Riyadh, Saudi Arabia; h Division of Translational Pathology, Department of Pathology, King Abdulaziz Medical City, Ministry of National Guard-Health Affairs (NGHA), Riyadh, Saudi Arabia; i Department of Pediatrics, College of Medicine, Qassim University, Buraidah, Saudi Arabia

**Keywords:** Dihydrolipoamide dehydrogenase deficiency, Lactic acidosis, Hypoglycemia, Pyruvate dehydrogenase complex, Flavoprotein and E3, DLDD, Dihydrolipoamide Dehydrogenase Deficiency, BCAAs, Branched Chain Amino Acids, WES, Whole Exome Sequencing, MRI, Magnetic resonance imaging, IRB, Institutional Review Board, KAIMRC, King Abdullah International Medical Research Centre, DCA, Dichloroacetate, BCKDH, Branched-chain a-keto acid dehydrogenase, αKGDH, alpha-ketoglutarate dehydrogenase, PDH, Pyruvate dehydrogenase

## Abstract

**Background:**

Dihydrolipoamide dehydrogenase deficiency (DLDD) is a rare metabolic disorder inherited in an autosomal recessive manner. This heterogeneous disease has a variable clinical presentation, onset, and biochemical markers.

**Materials and methods:**

We retrospectively reviewed the clinical and molecular diagnosis of eight cases with DLDD from four referral centers in Saudi Arabia.

**Results:**

Remarkably, we found hepatic involvement ranging from acute hepatic failure to chronic hepatitis in five patients. In addition, neurological disorders in the form of seizures, developmental delay, ataxia, hypotonia and psychomotor symptoms were found in five patients, two of them with a combination of hepatic and neurological symptoms. In addition, only one patient had recurrent episodes of hypoglycemia. While most patients had the hepatic form of homozygous variant c.685G > T in the *DLD* gene, one patient was found to have a novel variant c.623C > T that had neurological and hepatic symptoms**.**

**Conclusions:**

We describe the largest reported DLDD cohort in the Saudi population. Clinical, biochemical, radiological, and molecular characterization was reviewed and no clear genotype-phenotype correlation was found in this cohort.

## 1 Introduction

Dihydrolipoamide dehydrogenase deficiency (DLDD) is an extremely rare metabolic disorder with autosomal recessive inheritance [1]. The highest carrier rate for a pathogenic mutation of the *DLD* gene has been found in the Ashkenazi Jewish population (1:94 to 1:110, with a disease frequency of 1:35,000 to 1:48,000) [2]. The disease is less common in other populations, such as the Mediterranean population, although the exact incidence and carrier frequency are still unknown [3].

DLDD is caused by a deficiency of the enzyme the dihydrolipoamide dehydrogenase, which is encoded by the *DLD* gene in 7q31.1 [4]. This enzyme, a flavoprotein unit designated E3, is one of three mitochondrial multi-enzymatic complexes, which include a pyruvate dehydrogenase complex, an a-ketoglutarate dehydrogenase complex, and a branched-chain a-keto acid complex, and plays a role in the glycine cleavage system. [5]. The complexity of this enzyme and the multiple biochemical functions would explain the variability (clinical heterogeneity) in affected patients, with a remarkable variability in phenotype, onset, and severity [6]. Most cases occur in the first year of life, however neonates affected by DLDD also present with hypotonia, poor feeding, limb rigidity, and/or choreoathetoid movements [3], [7], [8]. Patients may suffer from recurrent episodes of encephalopathy triggered by infections, prolonged fasting, inadequate caloric intake and other catabolic stresses [9]. Previous reports have described exercise induced myopathy with persistent bilateral ptosis and lactic acidosis in some patients. An extremely rare case of myoglobinuria with a high creatinine kinase was observed in adults [10], [11], [12]. In addition, hepatocellular dysfunction and recurrent acute liver failure (Reye-like syndrome) have been well described in DLDD patients, associated with high transaminases, hepatomegaly, coagulopathy, and lactic acidosis [9], [12]. Some patients with DLDD may have a milder course with fewer or no decompensatory episodes [13]. In a two-day-old neonate, myocardial dysfunction was observed to occur rarely, whereas several episodes of hepatic encephalopathy were noted in childhood [13].

Biochemical parameters include hypoglycemia, lactic acidosis, and mild hyperammonemia. In addition, metabolic derangements such as high urinary pyruvate, organic acids with peak levels of 2-hydroxybutyric acid, 2-hydroxyisovaleric acid, glutaric acid, adipic acid, and 2-oxoglutaric acid, and moderate peak levels of 2-hydroxyglutaric acid and 4-hydroxyphenyllactic acid have been observed during acute episodes [9], [14]. DLDD patients were found to have high levels of branched-chain amino acids (BCAAs) in amino acid plasma, with elevated citrulline, which was recently identified as a marker in symptomatic patients with DLDD [7], [10], [15], [16]. MRI findings of the brain varied between abnormal signal intensity in different brain regions, including the basal ganglia brain stem, thalamus and/or frontal and occipital lobes, mild cortical atrophy with mild periventricular white matter abnormalities. Normal brain images have also been reported [10], [13], [17], [18].

Because there is no established genotype-phenotype correlation, it is difficult to predict the patient's phenotype based on the detected variant. The variability is directly related to the effects of the mutation on the activity of the enzymatic complex [10]. To date, there is no effective treatment, although the effect of dietary supplements on disease progression and the episode severity has been reported in the literature [19], [20], [21], [22].

We retrospectively reviewed the clinical and molecular diagnosis of 8 cases with a confirmed diagnosis of DLDD from four participating centers in Saudi Arabia to describe the phenotypic spectrum of this rare disease, which may help to establish a consensus on a treatment protocol for such cases in the future.

## 2 Materials and methods

### 2.1 Ethical study approval and patient consents

This study was approved by the Institutional Review Board (IRB) of the King Abdullah International Medical Research Centre (KAIMRC) (IRB Number: IRBC/0366/19).

### 2.2 Patient data

We reviewed the charts of pediatric patients from four major referral centers in Riyadh, Saudi Arabia, with a diagnosis of DLDD. Data were collected from collaborating physicians at the four participating centers in Saudi Arabia. All available clinical data, laboratory data, and MRI data of the brain to confirm the diagnosis of DLDD were obtained from the medical records.

## 3 Results

### 3.1 Clinical findings

Eight patients (four males and four females) from eight unrelated families diagnosed with DLDD based on clinical and molecular tests, were included in this study. The age of the patients ranged from 2 years to 18 years, with a mean age of 8.5 years. Consanguinity was found in one family (12.5%). Symptoms occurred between birth and late childhood. In one of the patients, symptoms occurred in the neonatal period, in three patients in infancy, and in four in early and late childhood. The clinical phenotype included neurological manifestations with mental retardation in five patients, episodes of hepatocellular dysfunction in five patients, two of them with combined hepatic and neurological presentation (patients 4 and 8). One patient with hepatic presentation (patient 1) had episodic ketotic hypoglycemia with lactic acidemia during mild illnesses. Most of these episodes were triggered by infections, prolonged fasting, inadequate caloric intake, and catabolic stress. Patients with a hepatic manifestation had at least one episode of hepatic encephalopathy that recovered completely and had normal or near-normal mental functions with the exception of one patient (patient 8). In addition, mild hypertrophic cardiomyopathy was observed in two patients (patients 2 and 7) (Table 1).

**Table 1:** Summery of patient demography, clinical, biochemical, molecular phenotypes.

Demography	Onset	Molecular test	Clinical phenotype	HCM	OFC	BCAA	LA	Brain images	Treatment
Patient 1 Male	2 years	Homozygous, c.685G > T p.(Gly229Cys) (Pathogenic)	Mild course with mild ketotic hypoglycemia, lactic acidosis, and mild persistent elevated AST / ALT during catabolic stress. He has normal psychomotor function with no neurological symptoms.	UK	N	Normal	Yes	UK	Carnitine, Coenzyme Q10, Thiamine, Riboflavin, and Biotin.
Patient 2 Female	16 years	Homozygous, c.685G > T p.(Gly229Cys) (Pathogenic)	Episodic cyclic vomiting with mild persistent elevated AST / ALT. She had two episodes of Reye-like syndrome. She has normal psychomotor function with no neurological symptoms.	Yes	N	Normal	Yes	Normal	Carnitine, Coenzyme Q10, Thiamine, Riboflavin, and Biotin.
Patient 3 Male	3 years	Homozygous, c.685G > T p.(Gly229Cys) (Pathogenic)	Recurrent acute hepatic failure, encephalopathy, with mild persistent elevated AST / ALT, associated with metabolic acidosis He has normal psychomotor function with no neurological symptoms	No	N	Normal	Yes	Normal	Carnitine, Coenzyme Q10, Thiamine, Riboflavin, and Biotin.
Patient 4 Female	5 months	Homozygous, c.685G > T p.(Gly229Cys) (Pathogenic)	She has psychomotor dysfunction (DD/ID, and seizures) with mild persistent elevated AST / ALT	No	Micro	Elevated	No	Bilateral and symmetrical increased T2-weighted signal intensity affecting the putamen and caudate nuclei with atrophic changes.	Antiepileptic: Levetiracetam Rehabilitation.
Patient 5 Male	1 year	Homozygous, c.685G > T p.(Gly229Cys) (Pathogenic)	She has psychomotor dysfunction and (DD/ID)	UK	Micro	Normal	No	Bilateral and symmetrical increased T2-weighted signal intensity affecting the putamen and caudate nuclei with atrophic changes.	Supportive
Patient 6 Female	At birth	Homozygous, c.1436A > T p.(Asp479Val) (Pathogenic)	She has psychomotor dysfunction (DD,/ID, seizures, and hypotonia).	No	N	Normal	Yes	Normal	Carnitine, Thiamine, Biotin and Dichloroacetate
Patient 7 Male	7 months	Homozygous, c.1436A > T p.(Asp479Val) (likely pathogenic)	She has psychomotor dysfunction (DD/ID, seizures (focal), hypotonia, dystonia and optic atrophy)	Yes, mild	N	Elevated	Yes	Thin corpus callosum	Carnitine, Riboflavin, Thiamine, Biotin, and Sodium bicarbonate
Patient 8 Female	15 months	Homozygous, c.623C > T p.(Ser208Phe) Variant of unknown significant)	She has psychomotor dysfunction (mild hypotonia and DD), Recurrent acute hepatic failure (Reye-like syndrome) and metabolic acidosis.	UK	N	Elevated	Yes	UK	Alpha Lipoic acid, Carnitine, Thiamine

(DD) developmental delay, (ID) intellectual disability, (HCM) hypertrophic cardiomyopathy, (UK) unknown, (OFC) head circumference, (BCAA) branched chain amino acid, (LA) lactic acidosis and (Micro) microcephaly. (ALT) Alanine transferase, (AST) Aspartate aminotransferase, (N) Normal.

### 3.2 Laboratory tests

Episodic lactic acidosis with metabolic acidosis occurred in six patients (75%), none of the cohort had sustained lactic acidosis but episodic elevations during decompensation, and hypoglycemia in only one case. Elevated levels of branched chain amino acids (BCAAs) with normal citrulline levels were observed in three patients (37%) with primary neurological presentations. No patients in this cohort underwent enzymatic testing.

### 3.3 Findings of images

**Fig. 1.**
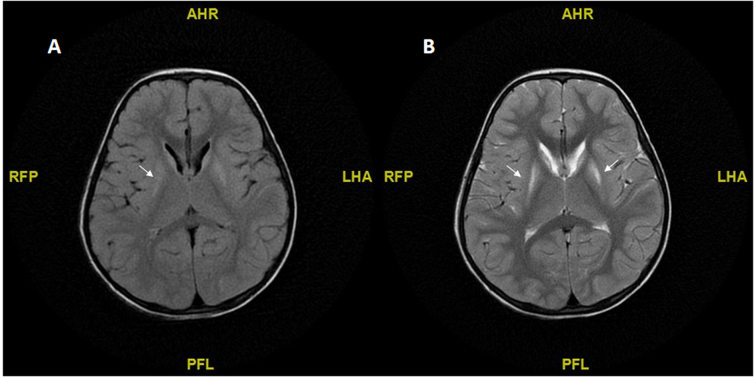
Fig 1a. MRI brain-FLAIR sequence. Fig 1b. MRI brain-T2 sequence Bilateral and symmetrical high signal intensity affecting putamen and caudate nuclei, the later is associated with atrophic changes.

MRI of the brain was performed in 6 of the patients. In the patients with isolated hepatic DLDD, the images showed a normal brain structure and normal myelination for age (2 patients). In the neurological DLDD group, the images varied between normal (1 patient), thin corpus callosum (1 patient), bilateral and symmetrical increased T2-weighted signal intensity involving the putamen and caudate nucleus with atrophic changes. [4], [5] (Fig. 1). None of the patients who underwent MRI examination of the brain had significant MRS findings.

### 3.4 Molecular genetic study data

Using Whole Exome Sequencing (WES), a homozygous pathogenic, probable pathogenic and a variant of unknown significance in the *DLD* gene were detected in all patients: NM_000108:3. The homozygosity inheritance related to the high consanguinity in our population. All variants detected were missense. The most frequently reported variant was c.685G > T, p.(Gly229Cys) which was detected in 5 patients (62%), mostly with hepatic form. In addition, c.1436A > T, p.(Asp479Val) was detected in two patients. Finally, one patient in this cohort has a novel variant c.623C > T, p.(Ser208Phe). This variant was not present in our local database, furthermore ACMGG classifies this variant as PM2, PP2, PP3. The substitution was classified as deleterious by computerized prediction tools (PolyPhen2, MutationTaster, FATHMM, PROVEAN, and SIFT).

## 4 Discussion

We reported the clinical phenotype, biochemical tests, and molecular tests of 8 patients with confirmed DLDD. The phenotype spectrum of this disease ranges from early-onset neurologic manifestations to late-onset liver involvement and, rarely, myopathic manifestations. The clinical and biochemical variability proved to be unpredictable on the sole basis of the genotypic characteristics or loss of enzymatic activity of the E3 component. This could be due to many factors related to the degree of increase in reactive oxygen species (ROS) especially during acidosis. The most frequently detected variant in the sample was c.685G > T p.(Gly229Cys), which was found in five patients (62%), as shown in (Table 1). In this group, the onset and clinical presentation were variable, ranging from early to late childhood. Both hepatic and neuropsychiatric DLDD were observed. Previously, the homozygous p.(Gly229Cys) variant was thought to be exclusively responsible for non-neurological manifestations, but later cases with early onset of neurological cases were reported with this variant, and milder phenotype could occur in the second decade of life [10], [23], [24]. Five cases (patients 1, 2, 3, 4, and 8) had hepatic manifestations in the form of episodic hepatic dysfunction, which may be associated with lactic acidosis, hypoglycemia and/or metabolic acidosis with coagulopathy. Three patients (patients 1, 2, and 8) developed hepatic encephalopathy (Reye-like syndrome) that recovered to baseline without significant neurologic complications. Interestingly, one patient (patient 2) diagnosed at age 13 after three episodes of acute hepatic encephalopathy within 6 months, showed dramatic improvement after being treated with nutritional supplements (Carnitine, Coenzyme Q10, Thiamine, Riboflavin, and Biotin). She was not hospitalized for three years after diagnosis and treatment. Neurological symptoms varied among the five patients, with two patients (patients 4 and 8) having a combination of hepatic and neurological presentation and abnormal neurological imaging findings when available. To date, the only affected DLDD patients with hepatic presentation who had neurological symptoms had experienced severe episodes associated with deep coma. This sometimes makes the distinction between the two types difficult and may explain the overlap of symptoms in cases 4 and 8.

In this cohort, one female patient (patient 6) had neonatal onset neurological manifestations due to c.1436A > T, p.(Asp479Val). The seizures occurred shortly after birth, were rapidly progressive, and were frequently recorded. She had microcephaly, developmental delay, mild behavioral disturbances, cerebellum signs, hypotonia, but no dystonia or cardiac involvement. Remarkably, her MRI scans were within normal structure and myelination for her age. Her clinical condition improved after the introduction of thiamine, biotin, carnitine, and dichloroacetate (DCA), which showed as a decrease in the number of crisis and admissions to the hospital. We discovered the same mutation in one patient in the current cohort (patient 7). His symptoms began at 7 months of age in the form of epilepsy, dystonia, optic atrophy, severe developmental delay, hypotonia, and a thin corpus callosum, in addition to an elevated BCAA levels, and lactic acidosis. Later in the course of the disease, he developed mild hypertrophic cardiomyopathy. This variant was reported in a patient with neonatal manifestations including episodes of apathy, metabolic acidosis, and lactic acidosis. At 9 months of age, he showed neurological symptoms in the form of developmental delay, hypotonia, microcephaly and mild hypertrophic cardiomyopathy [23].

Only one patient (patient 8) had a variant of unknown significance, c.623C > T p. (Ser208Phe), which had not been reported previously. This patient had mild developmental delay and hypotonia, showed episodic hepatic dysfunction, and metabolic dearangements (hyperammonemia, elevated BCAAs, high lactic acidosis (2.45–8.70 mmol/l), organic urine and was unremarkable. Elevated BCAAs occurred in three patients in this cohort (37%). Episodic lactic acidosis with metabolic acidosis in six patients (75%), and hypoglycemia in only two cases (25%). Quinonez et al. studied i the phenotype, biochemical markers, and genetic variant of 25 patients with different molecular outcomes in a larger cohort. Lactic acidosis was observed in 5 of the 25 patients (20%), elevated BCAA in 11 patients (44%), and hypoglycemia in only 4 patients (16%) [24]. To date, there are no consistent recommendations for the management of DLDD. In general, effective treatment depends not only on lowering the concentrations of pathological metabolites, but also on controlling the underlying metabolic derangement. Several strategies (e.g., restriction of proteins/BCAAs, oral DCA, thiamine, and lipoic acid supplementation, and nutritional therapy and consideration of a gastrostomy tube for persistent feeding problems) that have been tried in neurologic presentation do not appear to significantly alter disease progression. Lipoic acid is an essential cofactor for the E2 subunits of BCKDH, αKGDH, and PDH as well as to the glycine cleavage system. It has been reported in the literature that in some individuals with myopathic and hepatic presentation, laboratory values and/or clinical condition improved with supplementation alone or in combination with a low-protein diet [11], [20], [21]. In this cohort, most patients were started on supplementation with variable response. A systematic evaluation of the effect of available therapies is needed.

## 5 Conclusions

In this descriptive study, we describe the pattern of presentation and molecular characterization in eight DLDD patients. The correlation between genotype and phenotype cannot be predicted in DLDD patients. To date, there is no effective treatment for this disorder, so early detection, molecular confirmation, and prenatal testing are needed to prevent severe disease such as DLDD.

## Consent for publication

Informed consent to conduct the research study and to publish the cases was signed by the patients' fathers.

## Availability of data and materials

All data generated or analyzed as part of this study are included in this published article. Any additional data/files can be requested from the corresponding author.

## Authors' contributions

ALF, FAM prepared and summarized the literature, drafted the table, and wrote the manuscript. MAF, SAL, ALH, ITB, FAB, ALF edited the manuscript, collected the data, and contributed to the clinical diagnosis and management of the patients.

## Declaration of Competing Interest

The authors declare that they have no competing financial interests.
